# 

**Published:** 1879-06

**Authors:** 


VOL. XVIII. JUNE, 1879. NO. 11.

THE BUFFALO

MEDICAL AND SURGICAL JOURNAL.

